# Psychological Resilience after Hurricane Sandy: The Influence of Individual- and Community-Level Factors on Mental Health after a Large-Scale Natural Disaster

**DOI:** 10.1371/journal.pone.0125761

**Published:** 2015-05-11

**Authors:** Sarah R. Lowe, Laura Sampson, Oliver Gruebner, Sandro Galea

**Affiliations:** 1 Mailman School of Public Health, Columbia University, Department of Epidemiology, New York, New York, United States of America; 2 Boston University School of Public Health, Department of Epidemiology, Boston, Massachusetts, United States of America; University of California, San Francisco, UNITED STATES

## Abstract

Several individual-level factors are known to promote psychological resilience in the aftermath of disasters. Far less is known about the role of community-level factors in shaping postdisaster mental health. The purpose of this study was to explore the influence of both individual- and community-level factors on resilience after Hurricane Sandy. A representative sample of household residents (*N* = 418) from 293 New York City census tracts that were most heavily affected by the storm completed telephone interviews approximately 13–16 months postdisaster. Multilevel multivariable models explored the independent and interactive contributions of individual- and community-level factors to posttraumatic stress and depression symptoms. At the individual-level, having experienced or witnessed any lifetime traumatic event was significantly associated with higher depression and posttraumatic stress, whereas demographic characteristics (e.g., older age, non-Hispanic Black race) and more disaster-related stressors were significantly associated with higher posttraumatic stress only. At the community-level, living in an area with higher social capital was significantly associated with higher posttraumatic stress. Additionally, higher community economic development was associated with lower risk of depression only among participants who did not experience any disaster-related stressors. These results provide evidence that individual- and community-level resources and exposure operate in tandem to shape postdisaster resilience.

## Introduction

Hurricane Sandy made landfall as a post-tropical cyclone in the New York City (NYC) area on October 29, 2012, and led to $19 billion dollars in damages and 43 deaths in NYC [1]. To date, over $600 million has been allocated to rapid restoration of heat, power, and hot water service, and addressing NYC residents’ immediate needs [1]. An additional $3.22 billion in federal government funding has been granted to NYC for housing recovery efforts, infrastructural repairs, and investment in resiliency measures to withstand future natural disasters [1]. The substantial financial investment in infrastructural resilience after Hurricane Sandy paralleled a shift in research priorities from a focus on understanding postdisaster vulnerability toward understanding the factors that promote psychological resilience, defined as the ability to “bounce back” from disaster, sustaining low levels of psychological symptoms over time (e.g., [2]).

A large body of literature exploring the individual-level characteristics and disaster-related exposures that increase risk for adverse psychological outcomes, including posttraumatic stress disorder (PTSD) and major depression (MD), has provided insight into the factors that might produce resilience. Prior research, for example, has shown that individual-level indicators of socioeconomic disadvantage (e.g., race or ethnic minority status, unemployment), and exposure to more disaster-related traumatic events (e.g., bereavement) and stressors (e.g., prolonged displacement) are associated with greater odds of psychiatric disorder (e.g., [3, 4]). It follows that the converse of these factors—socioeconomic advantage and fewer disaster-related traumatic events and stressors—would be predictive of resilience.

Far less attention has been paid to the characteristics of communities that influence responses. This is an important limitation given that the resilience of individuals is inextricably linked to the resilience of the communities in which they live [5]. Community-level resources that are thought to promote resilience include *economic development*, or the volume, distribution and diversity of economic resources within a community, and *social capital*, which encompasses such constructs as received and perceived social support, sense of community, collective efficacy, and place attachment [6]. Conversely, greater levels of community-level disaster exposure are thought to hinder resilience [6]. Given the proposed interdependence of resilience at multiple levels, it is likely that community-level resources and exposure exert direct effects on individual-level psychological resilience, as well as influence the relationship between individual-level disaster exposure and resilience.

Only three published studies to our knowledge have used multilevel data to examine the role of these factors empirically [7–9]. These studies have provided preliminary evidence that community-level economic development, social capital and disaster exposure influence postdisaster psychological resilience and interact with individual-level factors in doing so. However, they are limited in their application to Hurricane Sandy in at least four ways. First, none of the studies took place in the context of a major metropolitan area like NYC. NYC provides an ideal geographic landscape for exploring community-level influences on resilience, given the broad variation in the economic and social characteristics of its many neighborhoods. Second, each study drew in part on aggregated self-report data to estimate community-level characteristics. Because only a portion of community residents participated in each study, these indices are perhaps not representative of the full community, and associations between community-level predictors and individual-level outcomes could be inflated. Third, the investigations focused exclusively on posttraumatic stress (PTS) and did not include symptoms of other disorders that have been found to be elevated in the aftermath of disasters, including MD. Lastly, none of the studies took into consideration the influence of individual-level exposure to additional traumatic events on postdisaster resilience. This is an especially important consideration in NYC, given that urban residents face greater exposure assaultive traumatic events than residents of other contexts [10], and that a previous study of NYC 9/11 survivors found that disaster exposure was not a significant predictor of long-term mental health, whereas exposure to community violence was [11].

This study therefore aimed to explore the influence of both individual- and community-level factors on postdisaster psychological resilience after Hurricane Sandy. We documented the prevalence of probable PTSD and probable MD 13–17 months postdisaster among a representative sample of residents in NYC communities that were most heavily affected by the storm. Subsequently, we examined associations between individual- and community-level factors and PTS and depression, and assessed whether community-level factors modified the influence of individual-level exposure to disaster-related stressors on outcomes.

## Material and Methods

### The Sample

Data were collected through telephone interviews with a random sample of New York City residents between December 2013 and March 2014. After the study was described to participants, oral consent from participants was obtained. Oral informed consent was employed instead of written informed consent because all interviews were conducted over the telephone. Interviewers documented participants’ informed consent in the study database. This approach to obtaining informed consent, as well as all other study procedures, was approved by the institutional review board of Columbia University. Interviewers for the study were employees at a survey research firm who each had prior training and experience in administering mental health measures via telephone. For the current study, they received additional training on the measures assessing PTS and depression symptoms, and conducted test-run interviews with experienced supervisors.

**Fig 1:**
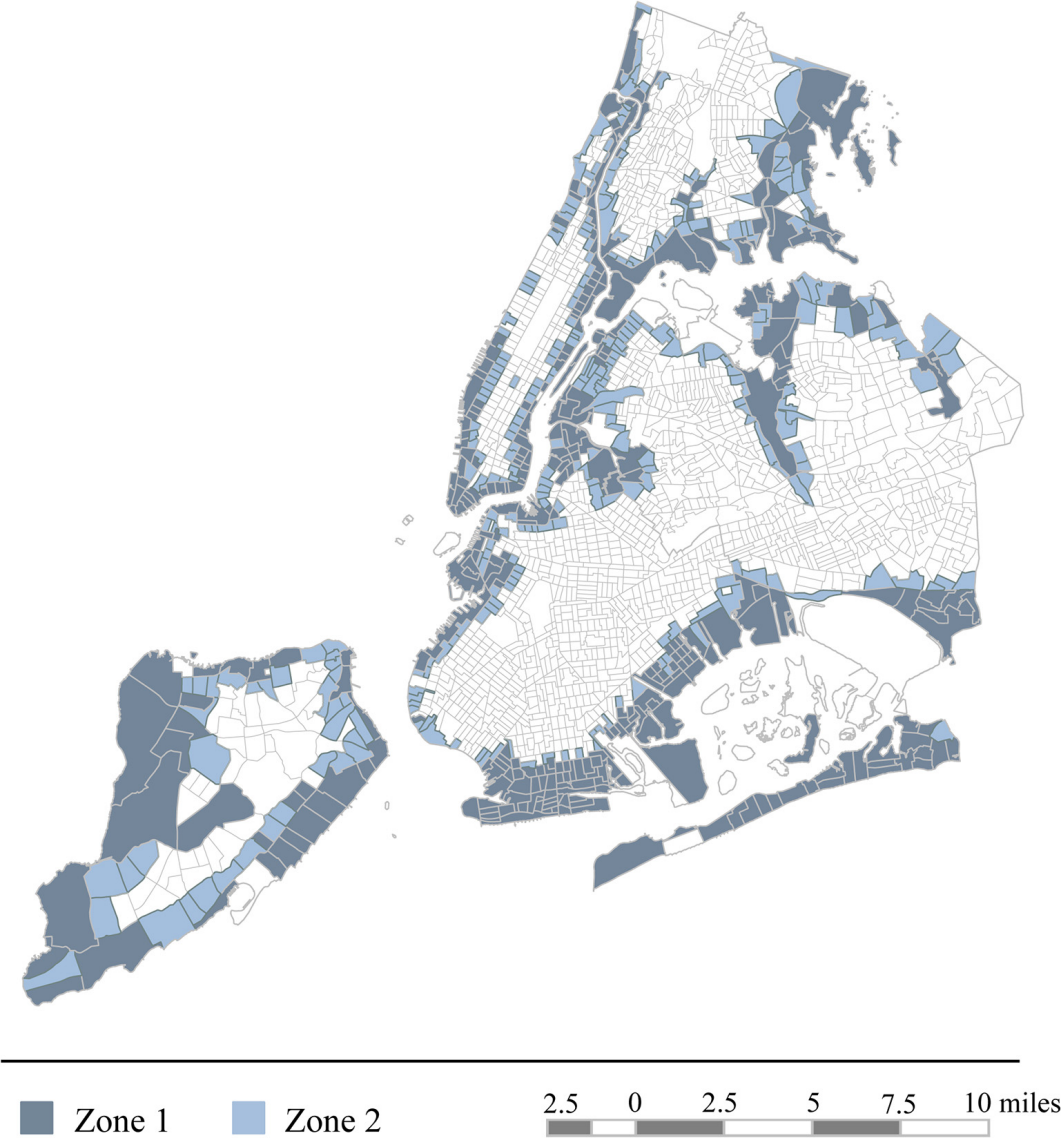
Map of Sampling Frame.

The sampling frame consisted of adults (18 years and older) with either landline or cellular telephones in Sandy-affected areas of New York City. Two sampling zones were derived based on flood inundation data from the FEMA Modeling Task Force (FEMA MOTF) [12]. Zone 1 consisted of census tracts in which 50% or more of the area was inundated with floodwater. Zone 2 consisted of census tracts in which some, but less than 50%, of the area was inundated and/or that were adjacent to the tracts from Zone 1 (Fig 1). Half of the sample within each zone was recruited via address-based sampling. Households were recruited by mail and telephone, and one adult was randomly selected from each household to participate. The other half was recruited through random-digit dialing of cellular phones, and a geographic screening determined whether participants whether potential participants were living in the sampling zones at the time of Hurricane Sandy. The overall response rate for the survey was 35% (American Association for Public Opinion Research [AAPOR] Cooperation Rate 2) [13]. The response rates for the address-based and cellular phone subsamples were 30% and 37%, respectively.

Of the 500 participants surveyed, 453 provided their pre-hurricane addresses, which were necessary for collecting community-level data. An additional 35 participants were excluded due to missing data. The final sample consisted of 418 participants who lived in 293 census tracts at the time of Hurricane Sandy. Demographic characteristics and other descriptive data are listed in Table 1.

**Table 1:** Means and Frequencies for All Variables in Included in the Study.

* *	*M / % (SE)*	*n*
*Individual-level*		
Age	46.06 (1.20)	—
Female	54.9% (3.3%)	260
Non-Hispanic Black	20.6% (2.7%)	78
Asian	6.1% (1.8%)	22
Hispanic	27.3% (3.2%)	80
Other race or ethnicity	9.4% (2.2%)	27
High school education or less	43.4% (3.4%)	106
Employed	50.7% (3.3%)	229
Parent, living with child at time of Sandy	26.3% (3.1%)	91
Married or cohabitating	46.6% (3.2%)	182
Experienced or witnessed trauma in addition to Sandy	44.9% (3.2%)	206
Number of disaster-related trauma	.06 (.02)	—
Number of disaster-related stressors	.69 (.08)	—
Posttraumatic stress (PCL-5 severity score)	7.25 (.71)	—
Depression (PHQ-9 severity score)	3.26 (.33)	—
*Community-level*		
Economic development [median household income]	$63,598.51 ($1,800.54)	—
Social capital [percentage of residents living alone]^a^	33.6% (.8%)	—
Disaster-related exposure [number of buildings affected, damaged, or destroyed]	121.48 (18.96)	—
Population density	19,324.54 (944.68)	—

*N* = 418 participants from 293 communities (census tracts).

^a^ A higher percentage of residents living alone was conceptualized as an inverse indicator of lower social capital. PCL-5 = Posttraumatic Stress Checklist for DSM-5. PHQ-9 = Patient Health Questionnaire-9.

### Measures

Participants completed a structured questionnaire in English or Spanish. They provided information on demographic characteristics, including their age, race and ethnicity, martial status, whether they were the parent or legal guardian of a child under 18-years old and living with this child at the time of the hurricane, level of education, and employment status at the time of the interview. They also reported whether they had experienced or witnessed any other traumatic events in addition to Hurricane Sandy in their lifetime.

Participants completed inventories of disaster-related traumatic events and stressors, with items drawn from other epidemiological surveys in the aftermath of major hurricanes [3, 4]. Disaster-related traumatic events included (a) whether the participant had been injured, (b) whether a close friend or family member had been injured, and (c) whether a close friend or family member had been killed, each as a direct result of the hurricane and its aftermath. A count of affirmative responses was computed. Disaster-related stressors included (a) whether the participant was displaced from their pre-hurricane home for over a week, (b) whether participant went without electricity, heat, or water for over a week, (c) whether there was damage to the participant’s pre-hurricane home, and (d) whether the participant experienced decline in income due to the hurricane and its aftermath. Again, a count of affirmative responses was computed.

Disaster-related posttraumatic stress (PTS) was assessed using the PTSD Checklist for DSM-5 (PCL-5) [14], a 20-item inventory measuring symptoms of PTSD as defined in the Diagnostic and Statistical Manual of Mental Disorders, Fifth Edition (DSM-5) [15]. Participants rated the extent to which they were bothered by each symptom in relation to Hurricane Sandy (e.g., “repeated, disturbing, and unwanted memories of Hurricane Sandy”) in the past 30 days from 0 (*not at all*) to 4 (*extremely*). All participants completed the PCL-5 irrespective of whether they reported a disaster-related traumatic event. Rather, the experience of Hurricane Sandy was considered the index trauma. Responses were summed to create a severity score, ranging from 0 to 80, and the recommended cutoff of 38 was used to define probable PTSD cases [14]. We note at the outset that screening scales are not substitutable for diagnostic assessments by a clinician. For this reason, we refer to participants as exceeding the recommended cutoff as having *probable* PTSD throughout. In doing so, we acknowledge the uncertainty regarding participants’ PTSD status inherent to how PTSD symptoms were measured in the study. Although the psychometric properties of the PCL-5 are under investigation, the DSM-IV version of the scale was shown to have excellent internal consistency and substantial agreement with PTSD diagnosis and symptom ratings, and its recommend cutoff score was shown to have a sensitivity of 0.94 and a specificity of 0.86 relative to the gold standard Clinician Administered PTSD Scale for DSM-IV [16]. Cronbach’s alpha reliability of the PCL-5 in the current study was 0.93.

Depression was assessed using the nine-item Patient Health Questionnaire (PHQ-9) [17]. Participants indicated how often over the past 30 days they had been bother by each symptom (e.g., “feeling down, depressed, or hopeless”) from 0 (*not at all*) to 3 (*nearly every day*), and a severity score was computed as the sum of all items, ranging from 0 to 27. A cutoff score of 10 was used to determine probable cases of major depression (MD), as prior assessment of criterion validity of the PHQ-9 has shown this cutoff to have a sensitivity of 0.88 and a specificity of 0.89 for MD relative to an independent structured interview by a mental health professional [18]. Again, since cases were not defined based on diagnostic assessments by a clinician, we refer to participants surpassing the cutoff has having *probable* MD throughout. Previous studies have found the PHQ-9 to have excellent internal consistency, test-retest reliability, and construct validity [18]. Cronbach’s alpha reliability of the PHQ-9 in the current study was 0.88.

Data on community-level economic development and social capital were collected from the 2007–2011 American Community Survey (ACS) five-year estimate, retrieved via Infoshare [19]. First, median household income was used as an indicator of economic development. Second, the percentage of participants living alone served as a proxy for social capital. Based on prior individual-level research showing negative associations between living alone and social capital (e.g., [20]), communities with more residents living alone were assumed to have lower social capital.

The number of buildings in each census tract that were affected, damaged, or destroyed served as an indicator of community-level disaster exposure. These values were computed in QGIS software [21] using a publically available geographic information systems (GIS) dataset from FEMA MOTF. The dataset contains point data on the level of damage to each building within the NYC flood inundation zone, which FEMA MOTF determined using information from Individual Assistance household inspections and building point damage determination estimates of visible aerial imagery. Because the impact of affected, damaged, or destroyed buildings could depend in part on the density of each community, we also included a control for population density. This was computed as the number of household residents in each tract, collected from the 2007–2011 ACS five-year estimate via Inforshare [19], divided by the area of each tract in square kilometers, calculated in QGIS.

### Statistical Analysis

We first calculated the prevalence of probable PTSD and probable MD in the sample. Next, we created maps in QGIS showing whether average levels of PTS and depression in each tract were in the low, medium, or high tertiles for the full sample, and whether community-level characteristics were in the low, medium, or high tertiles for the census tracts represented in the study. Bivariate associations between individual- and community-level factors and levels of PTS and depression were calculated in multiple multilevel regression models. Multilevel multivariable regression models were then computed, and cross-level interactions between disaster-related stressors and community-level factors were tested. All analyses were weighted to adjust for disproportionate sampling probabilities introduced by the sampling design and to correct for demographic differences between the sample and population. Data preparation and descriptive analyses were conducted in SPSS 21.0 [22] and multilevel analyses were conducted in Mplus 7.0 [23].

## Results

### Descriptive Information

The prevalence of probable PTSD was 2.0% (95% confidence interval [CI] = 0.9–4.3%), and the prevalence of probable MD was 8.9% (95% CI = 6.0–13.0%). Overall, 9.2% of participants met the criteria for either probable PTSD or probable MD (95% CI = 6.3–13.4%), and 1.7% met the criteria for both probable PTSD and probable MD (95% CI = 0.7–4.0%).

**Fig 2:**
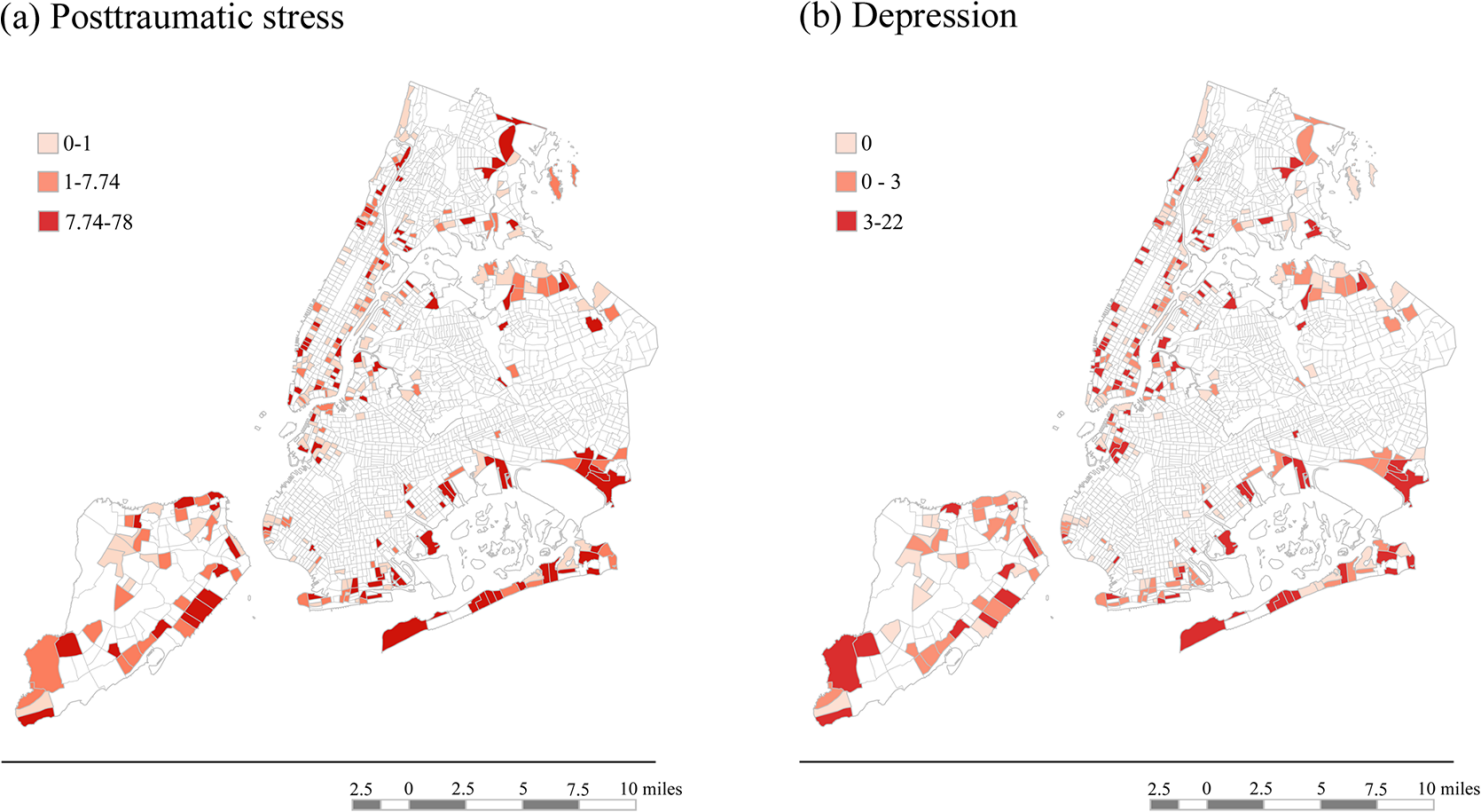
Maps of Mental Health Outcomes. Shading represents whether the average symptom scores for participants in each tract fell into the low, medium or high tertile of posttraumatic stress (Fig 2a) and depression (Fig 2b) for the sample.

**Fig 3:**
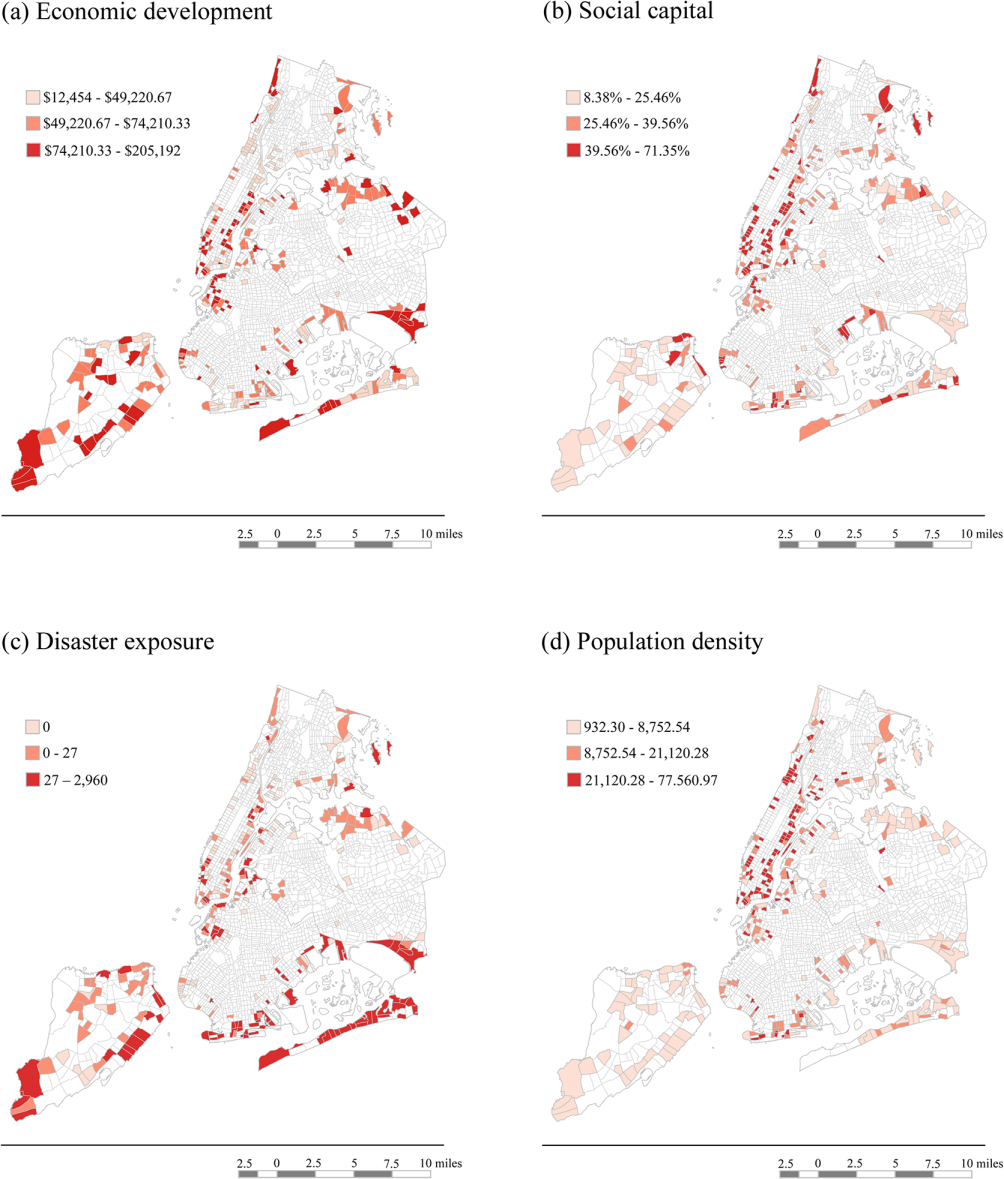
Maps of Community-Level Resources and Exposure. Shading represents whether the value for each tract fell into the low, medium or high tertile of economic development (medium household income; Fig 3a), social capital (percentage of residents living alone, with a higher percentage conceptualized as an indicator of lower social capital; Fig 3b), disaster exposure (number of buildings affected, damaged, or destroyed; Fig 3c), and population density (Fig 3d) for the tracts in which survey participants lived prior to Hurricane Sandy.

Fig 2 shows the geographic distribution of PTS and depression in the tracts represented in the study. We note here that, within our sampling zones, there were several tracts with no participants and that those tracts are therefore not filled in on the maps. It is also important to note that these maps are not representative of the census tracts as a whole, but rather reflective of the severity of each outcome across geographic space in our specific sample. In interpreting these maps, we noted that the tracts with PTS and depression in the high tertile were spread out across NYC’s five boroughs. Fig 3 shows the geographic distribution of the community-level factors in the census tracts represented by the study. Unlike the maps of outcomes, these maps are representative of the tracts as a whole, since they are based on community-level data from archival sources. We noted here that the tracts with economic development in the high tertile were spread were in each of the five boroughs. In contrast, the tracts with the percentage of residents living alone in the high tertile (i.e., with low social capital) and population density in the high tertile tended to be concentrated in Manhattan, and the tracts with disaster exposure in the high tertile were concentrated in the coastal areas of Staten Island and Queens.

### Bivariate Analyses

Table 2 shows the results of the multilevel bivariate analyses. Significant individual-level predictors of higher PTS included older age, non-Hispanic Black race, having a high school education or less, unemployment, and more disaster-related trauma and stressors. Significant community-level predictors were higher social capital (a lower percentage of residents living alone) and higher disaster exposure.

**Table 2:** Bivariate Associations between Individual- and Community-Level Factors and Posttraumatic Stress or Depression Symptoms.

* *	Posttraumatic Stress		Depression	
* *	*Estimate*	*SE*	*Estimate*	*SE*
*Individual-level*				
Age	.07**	.03	>-.01	.01
Female	1.40	1.13	-.01	.53
Non-Hispanic Black	5.47**	2.05	1.40	.73
Asian	-1.49	2.36	-.77	.98
Hispanic	2.09	1.35	.64	.66
Other race or ethnicity	-1.57	1.25	.20	.75
High school education or less	4.81**	1.47	1.35*	.62
Employed	-2.13*	1.02	-.51	.48
Parent, living with child at time of Sandy	.64	1.52	-.29	.64
Married or cohabitating	-1.18	1.09	-.52	.46
Experienced or witnessed trauma in addition to Sandy	1.78	1.12	1.45**	.50
Number of disaster-related trauma	3.97*	1.85	1.80*	.81
Number of disaster-related stressors	3.26***	.76	.87**	.29
*Community-level*				
Economic development [median household income]	< .01	< .01	< .01	< .01
Social capital [percentage of residents living alone]^a^	-.13**	.04	-.01	.02
Disaster-related exposure [number of buildings affected, damaged, or destroyed]	.01**	< .01	< .01	< .01
Population density	< .01	< .01	< .01	< .01

*N* = 418 participants in 293 communities (census tracts).

^a^ A higher percentage of residents living alone was conceptualized as an inverse indicator of lower social capital. *SE* = Standard Error. Results account for the multilevel data structure. Posttraumatic stress is indicated by Posttraumatic Checklist for DSM-5 (PCL-5) severity scores. Depression is indicated by Patient Health Questionnaire-9 (PHQ-9) severity scores.

* *p* < .05,

** *p* < .01,

*** *p* < .001

Significant individual-level predictors of higher depression were having experienced or witnessed any lifetime traumatic event other than Hurricane Sandy, and more disaster-related stressors. None of the community-level factors were significantly associated with depression.

### Multilevel Multivariable Analysis

Table 3 shows the results of the multilevel multivariable analyses. Significant individual-level predictors of higher PTS were older age, non-Hispanic Black race, Hispanic ethnicity, having experienced or witnessed any lifetime traumatic event other than Hurricane Sandy, and more disaster-related stressors. At the community-level, higher social capital (a lower percentage of residents living alone) was significantly associated with higher PTS.

**Table 3:** Results of Multilevel Multivariable Regression Models Predicting Posttraumatic Stress and Depression Symptoms.

	Posttraumatic Stress		Depression	
	*Estimate*	*SE*	*Estimate*	*SE*
*Individual-level*				
Age	.10**	.03	>-.01	.02
Female	1.10	1.12	.33	.56
Non-Hispanic Black	4.72*	1.83	1.20	.69
Asian	1.61	2.39	-.21	1.01
Hispanic	3.83**	1.23	1.02	.64
Other race or ethnicity	-1.74	1.26	.17	.78
High school education or less	2.29	1.36	1.15	.67
Employed	-1.12	1.08	-.46	.56
Parent, living with child at time of Sandy	1.11	1.39	-.19	.65
Married or cohabitating	-1.49	1.07	-.39	.49
Experienced or witnessed trauma in addition to Sandy	2.92*	1.15	1.67**	.52
Number of disaster-related trauma	.76	2.01	.87	.87
Number of disaster-related stressors	3.16***	.93	.72	.37
*Community-level*				
Economic development [median household income]	< .01	< .01	< .01	< .01
Social capital [percentage of residents living alone]^a^	-.07*	.04	.01	.02
Disaster exposure [number of buildings affected, damaged, or destroyed]	>-.01	< .01	< .01	< .01
Population density	< .01	< .01	< .01	< .01

*N* = 418 participants in 293 communities (census tracts).

^a^ A higher percentage of residents living alone was conceptualized as an inverse indicator of lower social capital. *SE* = Standard Error. Posttraumatic stress is indicated by Posttraumatic Checklist for DSM-5 (PCL-5) severity scores. Depression is indicated by Patient Health Questionnaire-9 (PHQ-9) severity scores.

* *p* < .05,

** *p* < .01,

*** *p* < .001

Having experienced or witnessed any lifetime traumatic event other than Hurricane Sandy was a significant individual-level predictor of higher depression. None of the community-level predictors of depression reached statistical significance.

**Fig 4:**
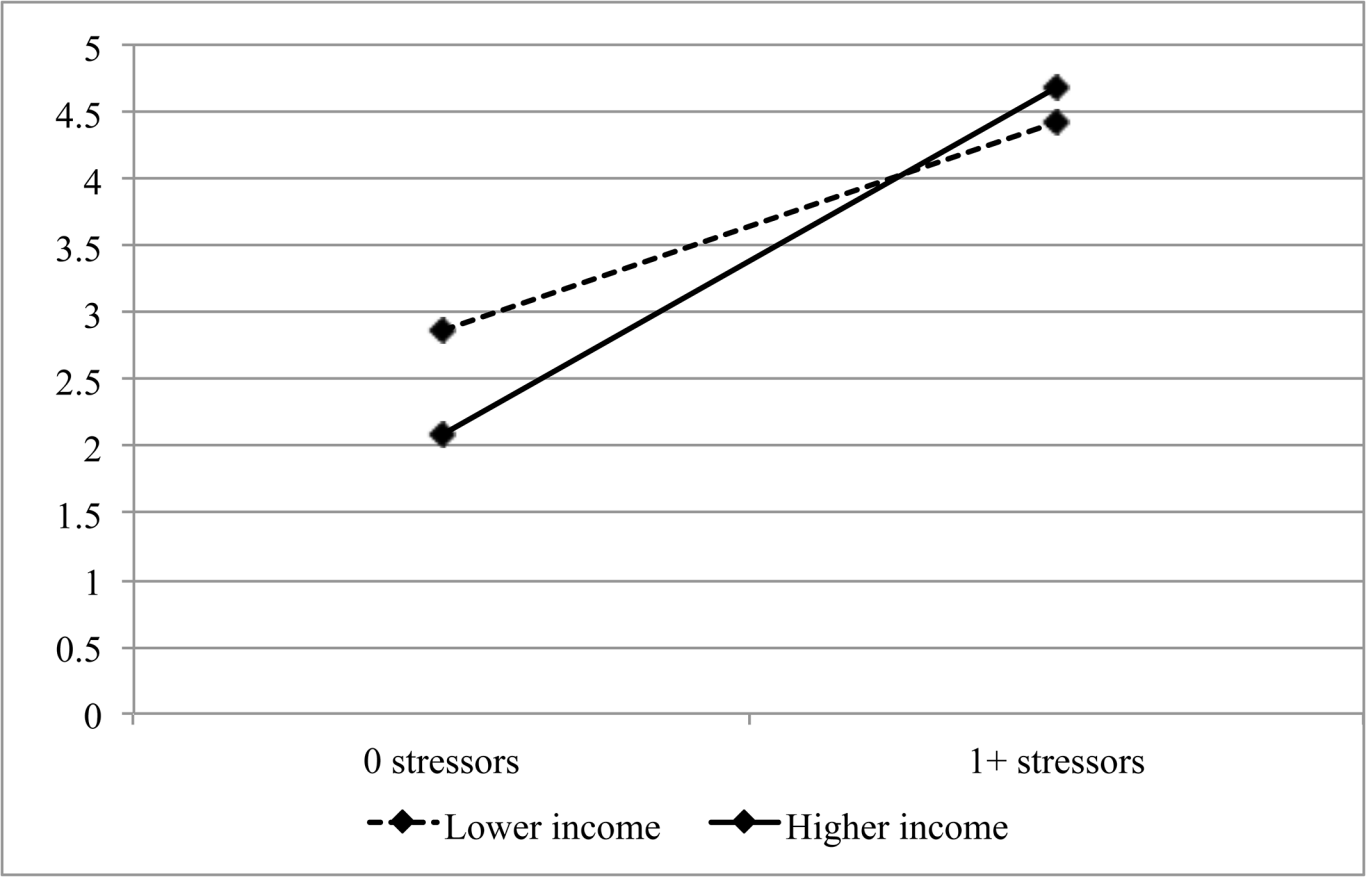
Illustration of the Cross-Level Interaction between Community-Level Economic Development and Individual-Level Disaster-Related Stessors. Economic development is indicated by median household income. The figure presents mean levels of depression computed with weighted individual-level data. *N* = 418.

The cross-level interaction between disaster-related stressors and economic development was significant (*Estimate [Est]* < .01, *Standard Error [SE]* < .01, *p* = .03). Fig 4 shows mean levels of depression for participants who had experienced no, or one or more, disaster-related stressors, living in communities with higher or lower economic development (based on median-split). This illustration suggested that economic development was negatively associated with depression only among participants who experienced no disaster-related stressors. Stratified multilevel analysis confirmed this interpretation. Among participants who had experienced no disaster-related stressors (*n* = 270), higher economic development was marginally associated with lower depression (*Est* >-.01, *SE* < .01, *p* = .07). Among participants who had experienced one or more disaster-related stressors (*n* = 148), economic development was not significantly associated with depression (*Est* < .01, *SE* < .01, *p* = .64).

## Discussion

We explored how a range of individual- and community-level factors related to PTS and depression symptoms among residents of NYC communities most heavily affected by Hurricane Sandy. The study yielded four key findings. First, the prevalence of probable PTSD and probable MD in the sample was 2.0% and 8.9%, respectively. Second, in multilevel multivariable models, significant individual-level predictors of higher PTS included demographic characteristics (e.g., older age, non-Hispanic Black race), a greater number of disaster-related stressors, and direct or witnessed exposure to another lifetime traumatic event in addition to Hurricane Sandy. In contrast, only directed or witnessed exposure to another lifetime traumatic event was associated with higher depression. Third, of the community-level factors included in the study, only one was significantly predictive of postdisaster psychiatric symptoms: residence in a community with higher social capital was significantly associated with higher PTS. Fourth, we found a significant cross-level interaction between community-level economic development and individual-level exposure to disaster-related stressors, such that living in a community with higher economic development was negatively associated with depression only for participants who did not endure any disaster-related stressors. Taken together, the findings provide additional evidence that community-level factors influence postdisaster resilience, both independently and in combination with individual-level stressor exposure.

This is the first study to our knowledge to document the prevalence of probable PTSD and probable MD in a representative sample exposed to Hurricane Sandy. As such, we are unable to compare the prevalence documented here to other Sandy-exposed samples. Compared to representative samples exposed to other natural disasters using similar measures to assess probable PTSD, the prevalence of probable PTSD is notably low. For example, a population-based study of southern Mississippi residents found the past-month prevalence of probable PTSD two years after Hurricane Katrina to be between 11.8 and 14.8% [24]. Another population-based study, of household residents exposed to Hurricane Ike, found the past-month prevalence of probable PTSD two to five months postdisaster to be 6.1% [4]. In contrast, the prevalence of probable MD in the current study was higher than in the Hurricane Ike study, wherein 4.9% were classified has having probable post-month MD [4]. Taken together, these results could indicate the NYC residents were remarkably resilient after Hurricane Sandy in terms of probable PTSD, but not in terms of probable MD, perhaps reflecting the psychological toll of ongoing stressors unrelated to the hurricane given the relatively low socioeconomic status of many areas hit by the hurricane [4]. However, these comparisons are made with caution in light of differences in the degree of damage and destruction rendered by each disaster, population demographics, and aspects of the study methodologies, including variation in timing of assessment and measures used to determine probable PTSD and probable MD cases.

The findings regarding individual-level predictors of PTS were generally consistent with those of prior research. We showed that the vulnerability of non-Hispanic Black and Hispanic persons and residents who experienced more disaster-related stressors documented in the aftermath of other disasters (e.g., [24, 25]) applies to Hurricane Sandy as well. Previous findings regarding the relationship between age and postdisaster mental health has been notably mixed [25], and the results suggest that older adults were at increased risk of higher PTS in this case. A notable divergence from prior research is that disaster-related traumatic events were not significantly associated with PTS in multivariable models. However, this could have been due to the relatively low incidence of such events in our sample—only 5.7% reported one or more disaster-related trauma—in comparison to other studies (e.g., [4]). In contrast, nearly half of participants (44.9%) reported having experienced or witnessed a lifetime traumatic event in addition to the hurricane, which was significantly associated with higher levels of both PTS and depression.

Unlike the majority of prior research on postdisaster mental health, we drew on archival data on community-level resources and hurricane exposure. The advantage of this approach, compared to aggregating self-report data across communities, was that the data better represented the communities as a whole and estimates of associations between community-level factors and mental health were more conservative. In contrast to our expectations, the only community-level factor to reach statistical significance in multilevel multivariable models was social capital, which was positively associated with PTS. Although we had conceptualized a lower percentage of residents living alone as an indicator of higher social capital, it might have been an inadequate proxy for the construct. It could be, for example, that a relatively *higher* percentage of residents living alone fosters greater connections between residents, thus leader to higher social capital. In exploration of the community-level data, we noted that only 40 of the 293 census tracts (13.7%) had more than 50% of participants living alone, and that the maximum percentage was 71.4%. It could be that, in our sample, a higher percentage of residents living alone served as a marker of residential diversity, and that communities with a mix of single persons, couples and families foster more opportunities of social connections. Alternatively, a higher percentage of residents living alone could indicate greater economic assets that were not captured by our measure of community-level economic development, median household income. In a similar vein, this construct could represent other aspects of community-level disaster exposure, which would be consistent with our observation that the tracts with a higher percentage of residents living alone were concentrated in Manhattan, whereas those with a greater disaster exposure were concentrated in the coastal areas of Queens and Staten Island.

The significant cross-level interaction between community-level economic development and individual-level disaster-related stressors was inconsistent with a prior finding indicating that community-level resources buffered against disaster exposure [9]. This divergence could be due to differences in the community resources and indicators of exposure included, and the different contexts in which the studies took place. Our result suggests that community economic resources might only confer benefits to residents who do not experience disaster-related stressors. One explanation could be that stressors interfered with residents’ ability to make use of community resources. For example, a displaced survivor might be less able to draw on resources from his community, compared to a survivor who was able to remain in his predisaster home.

The results of the study should be interpreted in light of its limitations. First, as noted previously, PTS and depression symptoms were assessed through self-report inventories, and probable PTSD and probable MD were defined by recommended cutoffs for these inventories rather than clinical interviews. Although this approach was time efficient and consistent with prior epidemiological studies in postdisaster contexts (e.g., [7]), the prevalences reported in this study should be interpreted as estimates at best, and future research employing diagnostic assessments by clinicians are needed to more accurately assess PTSD and MD in the aftermath of Hurricane Sandy. Second, participants reported on PTSD symptoms in reference to Hurricane Sandy regardless of whether they experienced a disaster-related traumatic event, as noted previously. The three disaster-related traumatic events assessed in the study did not encompass the full range of trauma exposures that participants might have experienced during the disaster. Lacking a thorough assessment of disaster-related trauma exposure, we did not limit our assessment of PTSD to trauma-exposed participants and, as such, the prevalence reported here, as well as those reported in prior epidemiological disaster studies sharing this limitation (e.g., [4, 24]), are likely underestimates. Third, interviews were conducted 13–16 months postdisaster and participants reported on symptoms experienced in the past 30 days. Prior research suggests that a substantial proportion of disaster survivors experience initially elevated symptoms that resolve within the first postdisaster year [26], and our assessment did not capture such responses. Fourth, our assessment of other experienced or witnessed lifetime trauma in addition to Hurricane Sandy was rough in that we did not information on the exact nature or timing of events. Although we were therefore unable to assess whether characteristics of other trauma related to postdisaster mental health, the rough assessment reduced the burden on participants and it was notable that this indicator, which could have included a broad variety of experiences, was a significant predictor of both mental health outcomes. Fifth, although our inclusion of community-level predictors surpassed that of previous studies, the factors included did not fully capture the full range of resources and exposures that could influence resilience. Sixth, census tracts might not reflect what residents perceive as their communities; however, the use of tracts has several advantages, such as the availability of tract-level data and stability of tract boundaries over time [27]. Seventh, our multilevel models did not assess the potential influence of the resources and exposures of neighboring or nearby communities on participants’ mental health. Eighth, although the overall response rate (35%) was consistent with other population-based studies in the field [28], it is worth noting that there may have been systematic differences between persons who opted to complete the study and those who did not and therefore potential for considerable participation bias. Further participation bias could have resulted from the administration of the survey over the telephone. For example, the results might not apply to residents of the sampling zone who either do not own telephones or who are less apt to answer calls from unfamiliar numbers or during the study’s recruitment times. Lastly, because the study was situated in NYC, the results might not generalize to other Sandy-affected areas or to communities exposed to other natural disasters.

Notwithstanding these limitations, this study provides evidence that individual- and community-level resources and exposure operate in tandem to shape postdisaster resilience. A further understanding of this issue will have broad implications for public health approaches to building resilience to natural disasters and other catastrophic events.
